# Urine anti-PLA2R antibody is a novel biomarker of idiopathic membranous nephropathy

**DOI:** 10.18632/oncotarget.19859

**Published:** 2017-08-03

**Authors:** Yu Wang, Yi-Xin He, Tian-Tian Diao, Shi-Yao Wei, Wen-Rui Qi, Cen-Cen Wang, Shu-Min Song, Min Bi, Chun-Mei Li, Cai-Xia Zhang, Yan-Pei Hou, Qiu-Ju Wei, Bing Li

**Affiliations:** ^1^ Department of Nephrology, Second Affiliated Hospital of Harbin Medical University, Harbin, People’s Republic of China; ^2^ Science and Technology Department, Financial Mathematics Major, Beijing Normal University, Hong Kong Baptist University United International College, Zhuhai, People’s Republic of China

**Keywords:** idiopathic membranous nephropathy, urine anti-PLA2R antibody, proteinuria, biomarker, diagnosis, Immunology and Microbiology Section, Immune response, Immunology

## Abstract

Since urine samples more directly reflect kidney alterations and damage than blood samples, we investigated whether urine anti-PLA_2_R antibody (uPLA_2_R-Ab) could be utilized similarly to serum anti-PLA_2_R antibody (sPLA_2_R-Ab) as a noninvasive biomarker of idiopathic membranous nephropathy (IMN). In this study, we performed a qualitative analysis using an indirect immunofluorescence test (IIFT) and measured uPLA_2_R-Ab and sPLA_2_R-Ab concentrations using an enzyme-linked immunosorbent assay (ELISA) in 28 patients with biopsy-proven IMN and 12 patients with secondary membranous nephropathy (SMN). Overall, 64.3% (*n*=18) of patients with IMN had IIFT-positive sPLA_2_R-Ab, 67.9% (*n*=19) of patients with IMN had IIFT-positive uPLA_2_R-Ab, and none of the SMN patients had IIFT-positive sPLA_2_R-Ab or uPLA_2_R-Ab. The titers of the anti-PLA_2_R antibody from the IMN patients in the urine (10.72±22.24 RU/μmol, presented as uPLA_2_R-Ab/urine creatinine) and serum (107.36±140.93 RU/ml) were higher than those from the SMN patients (0.51±0.46 RU/μmol, 0.008±0.029 RU/ml, respectively, *p*<0.05). Statistical analyses indicated that there were positive correlations between uPLA_2_R-Ab and gPLA_2_R, sPLA_2_R-Ab or urinary protein and negative correlations between uPLA_2_R-Ab and serum albumin in patients with IMN. In conclusion, uPLA_2_R-Ab is a novel biomarker of IMN. sPLA_2_R-Ab combined with uPLA_2_R-Ab might be more helpful for diagnosis and activity in PLA_2_R associated MN.

## INTRODUCTION

Membranous nephropathy (MN) is a major cause of nephrotic syndrome in adults [1-3], a leading cause of end-stage renal disease in patients with primary glomerulonephritis and the primary glomerulonephritis that recurs after kidney transplantation [4]. Although an accurate pathological diagnosis can be made using immunofluorescence, light microscopy and electron microscopy [5], the definitive pathophysiology of idiopathic membranous nephropathy remains unclear. In the early 2000s, neutral endopeptidase was identified as the first human podocyte antigen involved in neonatal alloimmune membranous nephropathy [6], and recent advances have been achieved toward an understanding of the pathophysiology of membranous nephropathy. Currently, IMN is considered an autoimmune disease due to the landmark discovery of the phospholipase A_2_ receptor (PLA_2_R), which was found to be targeted by circulating antibodies in 70%-80% of adult patients with IMN [7]. In addition to PLA_2_R, thrombospondin type-1 domain-containing 7A (THSD7A) is another relevant autoantigen, and circulating autoantibodies against this protein have been detected in 5-10% of patients who are negative for anti-PLA_2_R [8].

However, the exact mechanism of PLA_2_R-induced IMN is unknown. Genome-wide association studies have reported that the 6p21 *HLA-DQA1* and 2q24 *PLA*_*2*_*R1* loci are related to IMN in White European patients [9]. Furthermore, data from various cohorts in Europe [10, 11] and Asia [12-14] supported the observation that individuals who carried the risk alleles of the two genes described above tended to develop IMN.

Currently, tests to measure anti-PLA_2_R antibodies in the serum and to detect the PLA_2_R antigen in glomerular deposits can be performed routinely [4]. The following two standardized assays from EUROIMMUN are thought to be suitable for routine diagnostic purposes: an indirect immunofluorescence test (IIFT) and an enzyme-linked immunosorbent assay (ELISA) [5]. Many publications by us [15] and other researchers have confirmed the value of serum anti-PLA_2_R antibody (sPLA_2_R-Ab) and glomerular PLA_2_R (gPLA_2_R) in the diagnosis and monitoring of patients with IMN and the prediction of post-transplantation recurrence [10, 16-20]. A study that described a urine test for the early detection of kidney injury molecule-1 (Kim-1) inspired us to design an analogous test for the detection of the anti-PLA_2_R antibody in urine because urine samples more directly reflect kidney damage and alterations than do blood samples. Thus, the aim of this study was to determine whether urinary anti-PLA_2_R antibody (uPLA_2_R-Ab) levels could serve as a noninvasive indicator for the diagnosis of IMN and could reflect IMN activity and severity.

## RESULTS

### Clinical characteristics of patients with IMN or SMN

**Figure 1:**
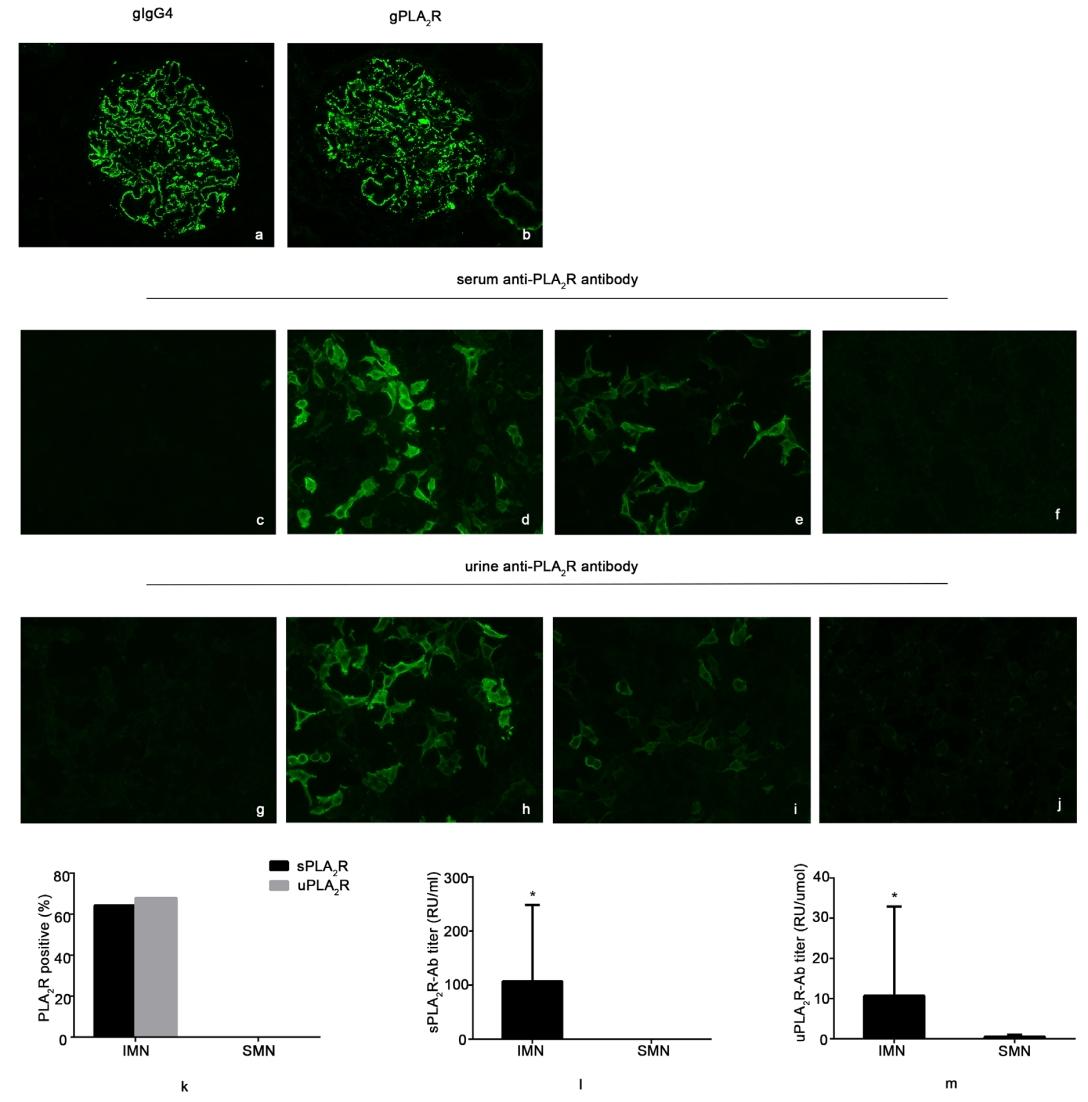
Detection of the expression of serum anti-PLA_2_R antibody (sPLA_2_R-Ab) and urine anti-PLA_2_R antibody (uPLA_2_R-Ab) by indirect immunofluorescence test (IIFT) and enzyme-linked immunosorbent assay (ELISA) **a.** and **b.** show one idiopathic membranous nephropathy (IMN) patient in this study who presented as glomerular IgG4- and PLA_2_R immunofluorescence positive. **c.** and **g.** show negative control biochips coated with cells that did not express the PLA_2_R protein that were incubated with serum and urine samples, respectively. **d.** and **h.** show the comparative fluorescence intensities of biochips that were incubated with serum and urine samples, respectively, from patient No. 23 in Supplementary Table 1. **e.** and **i.**, from patient No. 28, show higher fluorescence intensity in the section incubated with serum than with urine. **f.** and **j.**, from patient No. 36 with systemic lupus erythematosus, show samples that are negative for sPLA_2_R-Ab or uPLA_2_R-Ab. **k.** shows the positive expression of sPLA_2_R-Ab or uPLA_2_R-Ab in IMN and SMN patients by IIFT. **l.** and **m.** show the titers of sPLA_2_R-Ab or uPLA_2_R-Ab in IMN and SMN patients by ELISA. * *p* < 0.05

A total of 28 patients with biopsy-proven IMN and 12 patients with SMN were recruited in this study. We simultaneously collected serum and urine samples before biopsy. The renal pathology diagnosis referred to the immunofluorescence results of gPLA_2_R and IgG subtypes (Figures 1a-1b), as in our previous study [15]. All IMN patients were both gPLA_2_R- and IgG4-positive, but SMN patients were negative. Among the 12 SMN patients, 7 patients were diagnosed with systemic lupus erythematosus, 3 with connective tissue disease, and 2 with hepatitis B virus (HBV)-associated nephritis. As shown in Table 1, IMN patients were predominantly male, while more female patients (*p* < 0.05) were found in the SMN group. No significant differences in weight, age, proteinuria, serum albumin, serum creatinine or estimated glomerular filtration rate (eGFR) were found between IMN and SMN patients.

**Table 1:** Clinical characteristics and sPLA_2_R-Ab and uPLA_2_R-Ab titers of the IMN and SMN patients

Diagnosis	Number	Gender	Age	Weight (kg)	Urinary protein (g/24h)	Serum creatinine (μmol/L)	Serum albumin (g/L)	eGFR (ml/min/1.73m2)	sPLA2R-Ab titer (RU/ml)	uPLA2R-Ab titer (RU/ml)	Urine creatinine (μmol/L)	uPLA2R-Ab titer/ urine creatinine (RU/μmol)
IMN	28	male 17*	50.54±12.67	69.16±10.02	6.73±3.36	67.29±17.29	23.89±7.19	138.49±49.59	107.36±140.93**	94.61±187.57*	9276.79±5063.30	10.72±22.24*
SMN	12	male 2	40.17±7.25	62.79±14.14	7.83±7.34	71.63±26.60	22.73±8.15	122.07±41.03	0.01±0.03	3.36±2.38	8130.67±6262.25	0.51±0.46

IMN, idiopathic membranous nephropathy; SMN, secondary membranous nephropathy.

Differences in gender were observed between the two groups, and these characteristics may be associated with the etiologies of these two disease subtypes. Serum and urine PLA_2_R antibody titers as detected by ELISA in this study. To compare the urine ELISA results among the different samples, the uPLA_2_R-Ab ELISA results were adjusted to the urine creatinine results. ^**^ *p* < 0.01, ^*^ *p* < 0.05.

### uPLA_2_R-Ab is a good noninvasive indicator for IMN diagnosis

In this study, we determined that 64.3% (*n* = 18) of patients with IMN were sPLA_2_R-Ab-positive by qualitative analysis by IIFT. Urine more specifically reflects kidney injury than does blood. Thus, uPLA_2_R-Ab was examined in the present study. Surprisingly, 67.9% (*n* = 19) of patients with IMN were uPLA_2_R-Ab-positive (Figure 1k). Of these patients, 17 were simultaneously sPLA_2_R-Ab- and uPLA_2_R-Ab-positive. In contrast, no serum or urine sample from the SMN group was positive for the anti-PLA_2_R antibody (Figures 1c-1j). Furthermore, we performed quantitative analysis by ELISA (Table 1). The titers of sPLA_2_R-Ab (*p* < 0.01) and uPLA_2_R-Ab (*p* < 0.05) from the IMN group were significantly higher than those from the SMN patients (Figures 1l-1m).

### uPLA_2_R-Ab titer is highly correlated with sPLA_2_R-Ab level in patients with IMN

**Figure 2:**
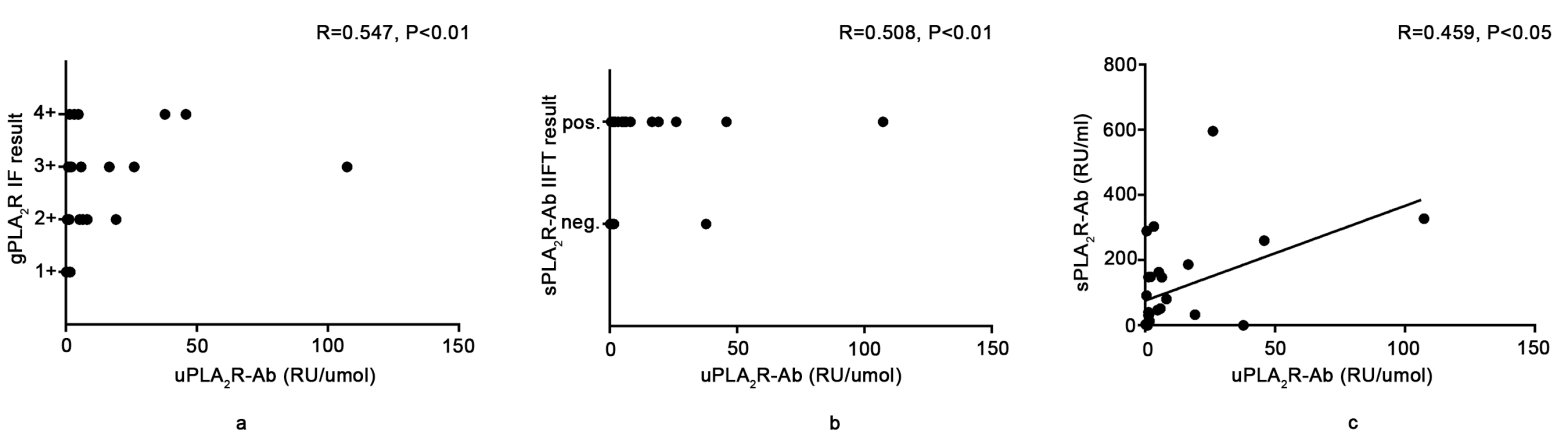
Relationship between the urine anti-PLA_2_R antibody titer and glomerular PLA_2_R antigen (gPLA_2_R) or serum anti-PLA_2_R antibody titer **a.** shows the positive relationship between gPLA_2_R IIFT intensity and the uPLA_2_R-Ab titer. **b.** and **c.** show the positive relationship between the sPLA_2_R-Ab IIFT intensity or titer and uPLA_2_R-Ab titer. To compare the uPLA_2_R-Ab titer from different patients, we adjusted the values to uPLA2R-Ab/urine creatinine.

We further investigated the association between the uPLA_2_R-Ab titer and gPLA_2_R intensity. As in our previous study, we divided gPLA_2_R IF results into four classes according to the immunofluorescence intensity [15]. To compare the uPLA_2_R-Ab titers of different patients, we adjusted the ELISA results to the urine creatinine from the same sample and presented the findings as uPLA_2_R-Ab titer/urine creatinine. As shown in Figure 2a, there was a positive correlation between gPLA_2_R intensity and the uPLA_2_R-Ab titer (*R* = 0.547, *p* < 0.01). As reported in our previous study, sPLA_2_R-Ab is a good noninvasive indicator of IMN activity and severity. In addition, monitoring the sPLA_2_R-Ab titer may assist in the determination of when to initiate the administration of immunosuppressive agents and in the evaluation of treatment efficacy [15]. Thus, we examined the correlation between the levels of uPLA_2_R-Ab and the levels of sPLA_2_R-Ab. As shown in Figures 2b-2c, there were significant positive correlations between the uPLA_2_R-Ab titer and the sPLA_2_R-Ab using both IIFT (*R* = 0.508, *p* < 0.01) and ELISA (*R* = 0.459, *p* < 0.05).

### uPLA_2_R-Ab is a good noninvasive biomarker for IMN activity and severity

**Figure 3:**
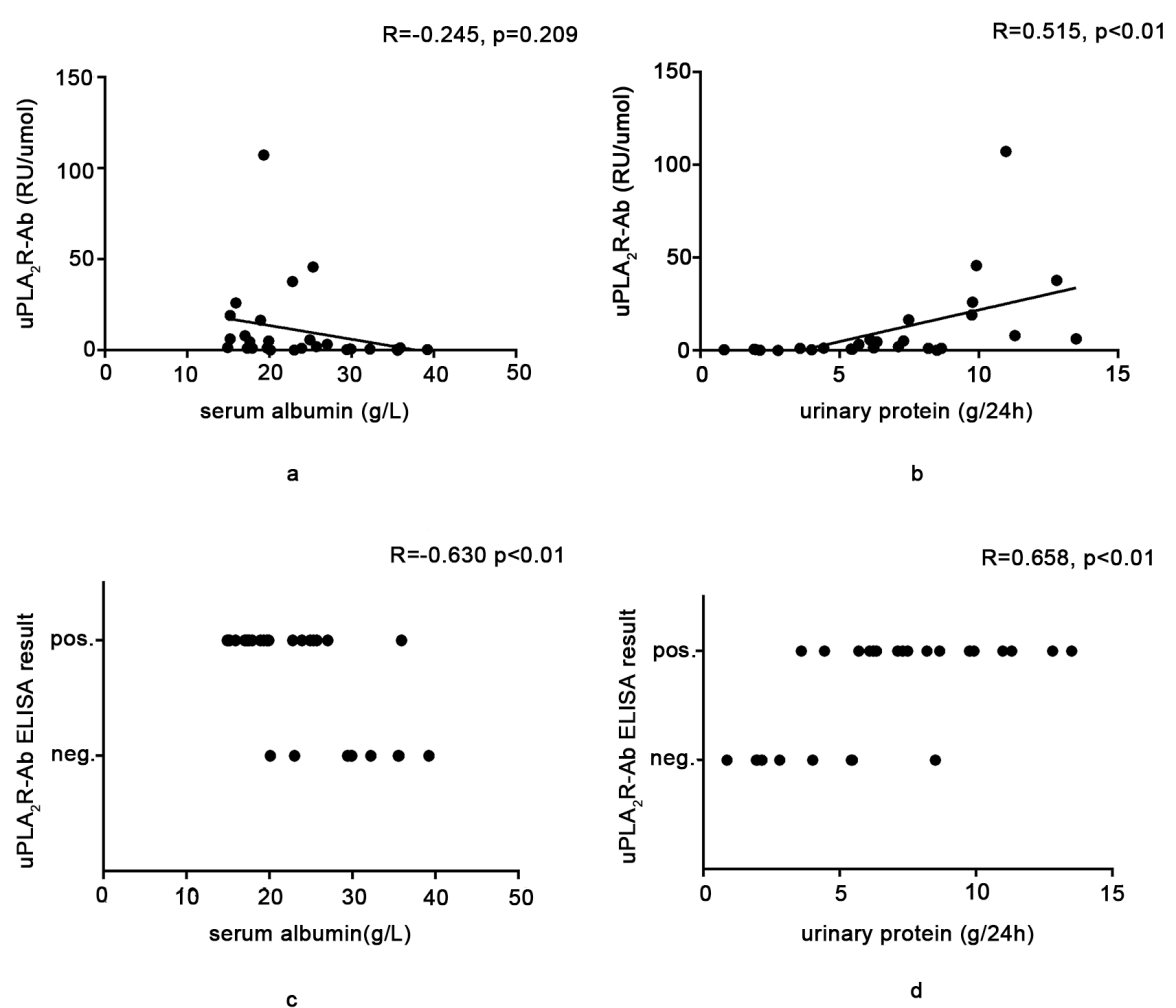
Relationship between the expression of the urine anti-PLA_2_R antibody and the clinical parameters **a.** and **b.** show the relationship between the uPLA_2_R-Ab titer, measured *via* ELISA, and the serum albumin level or proteinuria. A statistically significant positive relationship was found between the uPLA_2_R-Ab titer and proteinuria. If we defined the cutoff of uPLA2R-Ab/urine creatinine by ELISA as 1RU/μmol in **c.** and **d.**, we found that there were statistically significant relationships between the uPLA_2_R-Ab titer and the serum albumin level or proteinuria.

To assess whether the expression of uPLA_2_R-Ab can indicate IMN disease activity and severity similarly to sPLA_2_R-Ab [15], the relationship between uPLA_2_R-Ab and the clinical parameters was examined. The IIFT intensity of uPLA_2_R-Ab was positively correlated with proteinuria (*R* = 0.658, *p* < 0.01) and was negatively correlated with serum albumin (*R* = -0.573, *p* < 0.01). uPLA_2_R-Ab titer was positively correlated with proteinuria (*R* = 0.515, *p* < 0.01) but was not significantly correlated with albumin *R* = -0.245, *p* = 0.209) (Figures 3a-3b). We then observed that most uPLA_2_R-Ab/urine creatinine values in patients with IMN were > 1 RU/μmol, which was defined as a cutoff value in this study. We then divided patients into positive (uPLA_2_R-Ab titer/urine creatinine ≥1 RU/μmol) and negative groups (uPLA_2_R-Ab titer/urine creatinine < 1 RU/μmol). According to the rules above, the uPLA_2_R-Ab titer was positively correlated with proteinuria (*R* = 0.658, *p* < 0. 01) and was negatively correlated with serum albumin per ELISA (*R* = -0.630, *p* < 0.01) (Figures 3c-3d).

Taken together, these results indicate that uPLA_2_R-Ab may also be a good noninvasive biomarker for IMN activity and severity.

## DISCUSSION

The discovery of a serum anti-PLA_2_R antibody in patients with IMN has led to the clinical application of sPLA_2_R-Ab for diagnosis, disease activity monitoring, treatment response evaluations, and prognosis [7, 10, 16-20]. Because the evaluation of urinary indicators is a noninvasive and sensitive method for the estimation of proteinuria and for monitoring the therapeutic effects of an intervention, which would be a requirement for an ideal biomarker of IMN, we investigated whether a urinary anti-PLA_2_R antibody could be detected in MN patients. Experimental and human studies of IMN mechanisms have indicated that immunoglobulins along the glomerular basement membrane (GBM) initiate a sequence of events that include complement activation, an impaired sieving function of the glomerular capillary wall and the pore-forming slit diaphragm, and eventual proteinuria [21, 22]. In IMN, non-selective proteinuria leakage occurs through the impaired filtration barrier from blood to crude urine. The composition of the crude urine accordingly contains circulating sPLA_2_R-Ab, which crosses the impaired filtration barrier and does not bind gPLA_2_R. Thus, we speculated that an anti-PLA_2_R antibody should be found in urine and could be detected by ELISA and IIFT, as with sPLA_2_R-Ab reported before [15]. In our study, we found that the expression of uPLA_2_R-Ab was positively correlated with sPLA_2_R-Ab as detected *via* ELISA and IIFT. The urinary anti-PLA_2_R antibody titer was related to the proteinuria activity and severity and the expression of the serum anti-PLA_2_R antibody. These results confirmed that the urinary anti-PLA_2_R antibody could be a biomarker of IMN.

Many reports have assessed the sPLA_2_R antibody and the glomerular antigen (gPLA_2_R) and reported that sPLA_2_R-Ab and gPLA_2_R were not always found at the same time [23, 24]. This phenomenon was also observed in our previous study [15] and other publications [25-27]. Some patients who had a high level of serum anti-PLA_2_R autoantibodies were not PLA_2_R-positive in the glomerular deposits; in contrast, some patients had no detectable serum anti-PLA_2_R autoantibodies but were PLA_2_R-Ab-positive in the glomerular deposits. The possible reasons were this finding could be as follows: 1. The former phenomenon might reflect the early stage of MN, because the antibodies remained in circulation in the blood but were poorly accessible at the time of kidney biopsy, and the latter might reflect the recovery or inactive stage of MN. Therefore, the titer of the sPLA_2_R-Ab returned to normal, and the depositions in the glomeruli could not be cleared completely, resulting in gPLA_2_R positivity. 2. Urine samples more directly reflect kidney damage and deposit than blood samples. The inability to detect the sPLA_2_R-Ab, gPLA_2_R and uPLA_2_R-Ab simultaneously may reflect that the different stages of IMN. Therefore, sPLA_2_R-Ab combined with uPLA_2_R-Ab might be more helpful in the diagnosis and activity assessment of PLA_2_R-associated MN.

Notably, we observed that one patient with SMN (No.31) was uPLA_2_R-ab positive (Supplementary Table 2). Similar results were also reported in our previous study and in studies by other research groups [28, 29]. These authors found that a few patients with SMN were sPLA_2_R-Ab- or gPLA2R-positive. We believe that these patients most likely represent the occurrence of IMN and SMN; however, the precise mechanism remains unclear.

sPLA_2_R-Ab detection by IIFT and ELISA should be considered an advance compared with the immunofluorescence detection of gPLA_2_R because noninvasive serology tests that replace re-biopsy are more convenient and can be used to reflect the activity and to monitor the response to treatment. In particular, for some patients unable to undergo the biopsy, sPLA_2_R-Ab detection may replace biopsy to some extent. In our study, uPLA_2_R-Ab was similar to sPLA_2_R-Ab [15] and was positively correlated with proteinuria and negatively correlated with albumin. The titers of uPLA_2_R-Ab as detected *via* ELISA were parallel with the titers of sPLA_2_R-Ab as detected *via* ELISA. We used IIFT and ELISA to assess a 52-year-old male patient who had nephrotic range proteinuria and deep vein thrombosis who was unsuitable for biopsy. His serum and urine IIFT were positive and the sPLA_2_R-Ab and uPLA_2_R-Ab/urine creatinine titers were 109.397 RU/ml and 98.34 RU/μmol, respectively. Although we did not perform a biopsy for him, a definitive diagnosis of IMN could still be made, and the high PLA_2_R-Ab titers found in this patient’s blood and urine were consistent with his disease activity.

A recent study indicated that the evaluation of the levels of sPLA_2_R-Ab and the detection of gPLA_2_R deposition may provide additional information [30]. For instance, of the gPLA_2_R+ patients, the sPLA_2_R-Ab+ patients exhibited greater proteinuria and a lower chance of proteinuria remission than the gPLA_2_R- patients. Urine samples more directly reflect kidney alterations and damage than blood samples. In conclusion, uPLA_2_R-Ab is a novel biomarker of IMN. sPLA_2_R-Ab combined with uPLA_2_R-Ab might be more helpful in the diagnosis and activity assessment of PLA_2_R-associated MN.

## MATERIALS AND METHODS

### Patients and samples

In this study, 28 patients with biopsy-proven IMN and 12 patients with SMN who were diagnosed between 2015 and 2016 were enrolled from the Second Affiliated Hospital of Harbin Medical University. The 12 SMN patients included 7 who were diagnosed with systemic lupus erythematosus, 3 who were diagnosed with connective tissue disease, and 2 who were diagnosed with HBV-associated nephritis. The pathologic diagnosis of membranous nephropathy was made by light microscopy, immunofluorescence and electron microscopy.

Diagnosis of IMN was established by the following criteria [31]: Kidney biopsy with evidence of immune and electron-dense sub-epithelial deposits, without any deposition in sub-endothelial or mesangial areas. NO glomerular infiltrating cells or proliferation of mesangial and endothelial cells. IgG deposition, with a granular pattern, along the glomerular capillary walls (in association or not with the C3 complement fraction) proven by immunofluorescence. Secondary causes of MN were excluded after extensive clinical workup including detailed medical history and patient examination, serological markers of systemic autoimmunity such as anti-nuclear anti-bodies, anti-dsDNA, anti-ribonucleoproteins, anti-Smith, anti-Ro (SSA), anti-Ro (SSB), anti-topoisomerase I (SCI-70), anti-centromere, anti-Jo-l, anti-cardiolipin, anti-β2-glycoprotein I antibodies and thyroid autoimmunity. Viral hepatitis B and C and HIV infections, neoplastic conditions and exposure to toxic agents were also ruled out.

### Measurement of sPLA_2_R-Ab and uPLA_2_R-Ab

Blood and urine samples of all enrolled patients were collected at different time points. sPLA_2_R-Ab and uPLA_2_R-Ab were measured in all patients with an indirect immunofluorescence test (EUROIMMUN AG, Lübeck, Schleswig-Holstein, Germany) and an ELISA test (EUROIMMUN AG, Lübeck, Schleswig-Holstein, Germany) according to the manufacturer’s instructions. The serum ELISA results were considered positive at a level of > 20 RU/ml. Although there is no recommended cutoff value or dilution ratio for the urine ELISA, we attempted different dilution ratios (including 1:10, 1:50, 1:100, 1:200, and 1:1000). Finally, we found that when the urine samples were diluted 1:10, uPLA_2_R-Ab could be detected in the samples. To compare the urine ELISA results from different samples, we adjusted the urine ELISA results to the urine creatinine values of the same sample. Most uPLA_2_R-Ab/urine creatinine values were > 1 RU/μmol in patients with IMN, so we defined this as a cutoff value in this study for the statistical analysis; whether this value indicates two subtypes needs further research.

### Statistical analyses

All analyses were performed using SPSS 19.0 software. The data are expressed as the means ± SD. Distributions between groups were assessed using the chi-square test and *T*-test; differences of the serum or urine titers by ELISA between the IMN and SMN group were analyzed with *T*-test; agreement between sPLA_2_R-Ab and uPLA_2_R-Ab and the correlations between proteinuria, albumin and uPLA_2_R-Ab were analyzed with Pearson or Spearman’s rank correlation coefficients.

## SUPPLEMENTARY MATERIALS TABLES





## Abbreviations

(uPLA2R-Ab): urine anti-PLA2R antibody
(sPLA2R-Ab): serum anti-PLA2R antibody
(IIFT): indirect immunofluorescence test
(ELISA): enzyme-linked immunosorbent assay
(SMN): secondary membranous nephropathy
(IMN): Idiopathic Membranous Nephropathy
(THSD7A): thrombospondin type-1 domain-containing 7A
(gPLA2R): glomerular PLA2R
(Kim-1): kidney injury molecule-1
(HBV)-associated nephritis: hepatitis B virus
(eGFR): estimated glomerular filtration rate
(GBM): glomerular basement membrane
