# Supplementary material for: Urine anti-PLA2R antibody is a novel biomarker of idiopathic membranous nephropathy
Source: Oncotarget. 2017 Aug 3;9(1):67–74. doi: 10.18632/oncotarget.19859 (PMC5787499; doi:10.18632/oncotarget.19859)
Supplement: Supplementary file 1 [file oncotarget-09-67-s001.docx]

**Supplementary Table 1: Clinical characteristics of the IMN and SMN patients in this study.**

| **No.** | **Gender** | **Age** | **Weight(kg)** | **Urinary protein (g/24h)** | **Serum creatinine (μmol/L)** | **Serum albumin (g/L)** | **eGFR (ml/min/1.73m^2^)** | **Pathology diagnosis** |
| --- | --- | --- | --- | --- | --- | --- | --- | --- |
| 1 | male | 58 | 72.5 | 9.78 | 97.3 | 15.9 | 90.06 | IMN |
| 2 | male | 51 | 70 | 2.145 | 60 | 23 | 161.49 | IMN |
| 3 | male | 44 | 75 | 7.3 | 71.3 | 19.9 | 136.36 | IMN |
| 4 | male | 42 | 65 | 5.46 | 57 | 29.9 | 178.23 | IMN |
| 5 | male | 39 | 75 | 12.8 | 75 | 22.8 | 131.82 | IMN |
| 6 | male | 69 | 69 | 6.22 | 67.5 | 14.9 | 132.58 | IMN |
| 7 | male | 46 | 88 | 1.98 | 58 | 35.5 | 171.49 | IMN |
| 8 | male | 60 | 95 | 5.7 | 83.2 | 27 | 107.15 | IMN |
| 9 | male | 41 | 60 | 4.437 | 61.7 | 19.7 | 163.45 | IMN |
| 10 | male | 25 | 74 | 8.66 | 74 | 17.3 | 146.52 | IMN |
| 11 | male | 59 | 60 | 6.1 | 87 | 24.9 | 102.12 | IMN |
| 12 | male | 42 | 81 | 2.8 | 67 | 35.6 | 147.9 | IMN |
| 13 | male | 62 | 75 | 1.938 | 87 | 32.2 | 101.09 | IMN |
| 14 | male | 47 | 75 | 7.49 | 80 | 18.9 | 117.81 | IMN |
| 15 | male | 58 | 51.5 | 8.2 | 76 | 23.9 | 119.77 | IMN |
| 16 | male | 60 | 82 | 11.3 | 61.7 | 17 | 151.29 | IMN |
| 17 | male | 69 | 65 | 6.348 | 82.2 | 17.6 | 105.62 | IMN |
| 18 | female | 55 | 60 | 8.5 | 43.2 | 20.1 | 140.58 | IMN |
| 19 | female | 44 | 75 | 3.59 | 30.2 | 17.9 | 272.67 | IMN |
| 20 | female | 49 | 54 | 5.42 | 56 | 29.8 | 130.82 | IMN |
| 21 | female | 43 | 65 | 6.24 | 49 | 35.9 | 156.71 | IMN |
| 22 | female | 21 | 70 | 0.86 | 32 | 39.2 | 296.37 | IMN |
| 23 | female | 74 | 65 | 10.98 | 84 | 19.3 | 75.35 | IMN |
| 24 | female | 50 | 65 | 9.75 | 101 | 15.2 | 65.96 | IMN |
| 25 | female | 49 | 60 | 13.5 | 59.3 | 15.2 | 122.45 | IMN |
| 26 | female | 39 | 55.5 | 9.924 | 61 | 25.3 | 124.14 | IMN |
| 27 | female | 49 | 70 | 4 | 61 | 29.4 | 118.52 | IMN |
| 28 | female | 70 | 64 | 7.12 | 61.4 | 25.7 | 109.42 | IMN |
| 29 | male | 40 | 85 | 1.7 | 101.7 | 35.7 | 92.28 | HBV-SMN |
| 30 | female | 32 | 51 | 11.2 | 53.3 | 25.3 | 151.01 | HBV-SMN |
| 31 | male | 47 | 80 | 18.29 | 53 | 15 | 189.46 | CTD-SMN |
| 32 | female | 37 | 65 | 8.66 | 85 | 17.1 | 85.56 | CTD-SMN |
| 33 | female | 48 | 61 | 1.01 | 58.3 | 27.3 | 125.4 | CTD-SMN |
| 34 | female | 34 | 41 | 0.702 | 59 | 24.1 | 132.66 | SLE-SMN |
| 35 | female | 39 | 60 | 14.4 | 137 | 13.9 | 48.8 | SLE-SMN |
| 36 | female | 47 | 55 | 0.7 | 58 | 32.5 | 126.69 | SLE-SMN |
| 37 | female | 25 | 70 | 11.72 | 50.3 | 10.9 | 169.74 | SLE-SMN |
| 38 | female | 44 | 41 | 3.2 | 66 | 22.5 | 110.61 | SLE-SMN |
| 39 | female | 48 | 69.5 | 20.79 | 88 | 16.4 | 77.98 | SLE-SMN |
| 40 | female | 41 | 75 | 1.627 | 50 | 32.1 | 154.59 | SLE-SMN |

HBV-SMN, Hepatitis B virus-associated secondary membranous nephropathy; CTD-SMN, connective tissue disease-associated secondary membranous nephropathy; SLE-SMN, systemic lupus erythematosus-associated secondary membranous nephropathy.

The clinical characteristics of the 40 patients in the entire study cohort, including patients with idiopathic membranous nephropathy (IMN) and secondary membranous nephropathy (SMN). Differences in gender were observed between the two groups (p<0.05), and these characteristics may be associated with the etiologies of these two disease subtypes.
