# Supplementary material for: Urine anti-PLA2R antibody is a novel biomarker of idiopathic membranous nephropathy
Source: Oncotarget. 2017 Aug 3;9(1):67–74. doi: 10.18632/oncotarget.19859 (PMC5787499; doi:10.18632/oncotarget.19859)
Supplement: Supplementary file 2 [file oncotarget-09-67-s002.docx]

**Supplementary Table 2****: sPLA_2_R-Ab and uPLA_2_R-Ab titers as detected by ELISA.**

| **No.** | **sPLA_2_R-Ab titer (RU/ml)** | **uPLA_2_R-Ab titer (RU/ml)** | **Urine creatinine (μmol/L)** | | **uPLA_2_R-Ab titer/urine creatinine (RU/μmol)** | |
| --- | --- | --- | --- | --- | --- | --- |
| 1 | 596.018 | 34.001 | | 1306 | | 26.03 |
| 2 | 3.289 | 0.817 | | 5297 | | 0.15 |
| 3 | 163.57 | 66.502 | | 12820 | | 5.19 |
| 4 | 0 | 10.749 | | 15711 | | 0.68 |
| 5 | 0 | 792.306 | | 20958 | | 37.8 |
| 6 | 12.69 | 12.684 | | 7812 | | 1.62 |
| 7 | 289.431 | 4.014 | | 8068 | | 0.5 |
| 8 | 303.886 | 53.292 | | 16189 | | 3.29 |
| 9 | 32.3 | 20.581 | | 15060 | | 1.37 |
| 10 | 148.128 | 11.26 | | 9395 | | 1.2 |
| 11 | 51.884 | 52.512 | | 9085 | | 5.78 |
| 12 | 0 | 0.511 | | 13690 | | 0.04 |
| 13 | 0 | 3.09 | | 4776 | | 0.65 |
| 14 | 187.104 | 59.813 | | 3612 | | 16.56 |
| 15 | 30.856 | 8.3 | | 6688 | | 1.24 |
| 16 | 81.079 | 142.476 | | 17540 | | 8.12 |
| 17 | 46.674 | 52.048 | | 11096 | | 4.69 |
| 18 | 0 | 1.286 | | 8164 | | 0.16 |
| 19 | 40.49 | 8.776 | | 7074 | | 1.24 |
| 20 | 0 | 3.548 | | 4078 | | 0.87 |
| 21 | 8.907 | 11.408 | | 7670 | | 1.49 |
| 22 | 0 | 2.529 | | 5244 | | 0.48 |
| 23 | 327.488 | 426.205 | | 3973 | | 107.28 |
| 24 | 33.313 | 328.417 | | 17142 | | 19.16 |
| 25 | 147.562 | 36.918 | | 5823 | | 6.34 |
| 26 | 260.289 | 492.866 | | 10767 | | 45.78 |
| 27 | 91.824 | 2.529 | | 5893 | | 0.43 |
| 28 | 149.267 | 9.678 | | 4819 | | 2.01 |
| 29 | 0.1 | 1.981 | | 10505 | | 0.19 |
| 30 | 0 | 7.632 | | 21894 | | 0.35 |
| 31 | 0 | 5.582 | | 3082 | | 1.81 |
| 32 | 0 | 2.363 | | 5013 | | 0.47 |
| 33 | 0 | 2.308 | | 5257 | | 0.44 |
| 34 | 0 | 1.981 | | 2758 | | 0.72 |
| 35 | 0 | 4.487 | | 14853 | | 0.3 |
| 36 | 0 | 1.764 | | 3476 | | 0.51 |
| 37 | 0 | 0.31 | | 3495 | | 0.09 |
| 38 | 0 | 0.562 | | 3476 | | 0.16 |
| 39 | 0 | 4.848 | | 15475 | | 0.31 |
| 40 | 0 | 6.526 | | 8284 | | 0.79 |

Serum and urine PLA_2_R antibody titers as detected by ELISA in this study. The results of serum ELISA were considered positive at a level of >20 RU/ml according the manufacturer’s instructions. To compare urine ELISA results from different samples, the uPLA_2_R-Ab ELISA results were adjusted to urine creatinine.
